# Identification of genetic loci shared between schizophrenia and the Big Five personality traits

**DOI:** 10.1038/s41598-017-02346-3

**Published:** 2017-05-22

**Authors:** Olav B. Smeland, Yunpeng Wang, Min-Tzu Lo, Wen Li, Oleksandr Frei, Aree Witoelar, Martin Tesli, David A. Hinds, Joyce Y. Tung, Srdjan Djurovic, Chi-Hua Chen, Anders M. Dale, Ole A. Andreassen

**Affiliations:** 1 NORMENT, KG Jebsen Centre for Psychosis Research, Institute of Clinical Medicine, University of Oslo and Division of Mental Health and Addiction, Oslo University Hospital, 0407 Oslo, Norway; 2 Department of Neurosciences, University of California San Diego, La Jolla, CA 92093 United States of America; 3 Department of Radiology, University of California, San Diego, La Jolla, CA 92093 United States of America; 4 Lovisenberg Diakonale Hospital, 0456 Oslo, Norway; 5 23andMe, Inc., Mountain View, CA 94041 United States of America; 6 Department of Medical Genetics, Oslo University Hospital, Oslo, Norway; 7 NORMENT, KG Jebsen Centre for Psychosis Research, Department of Clinical Science, University of Bergen, Bergen, Norway; 8 Department of Psychiatry, University of California, San Diego, La Jolla, CA USA

## Abstract

Schizophrenia is associated with differences in personality traits, and recent studies suggest that personality traits and schizophrenia share a genetic basis. Here we aimed to identify specific genetic loci shared between schizophrenia and the Big Five personality traits using a Bayesian statistical framework. Using summary statistics from genome-wide association studies (GWAS) on personality traits in the 23andMe cohort (n = 59,225) and schizophrenia in the Psychiatric Genomics Consortium cohort (n = 82,315), we evaluated overlap in common genetic variants. The Big Five personality traits neuroticism, extraversion, openness, agreeableness and conscientiousness were measured using a web implementation of the Big Five Inventory. Applying the conditional false discovery rate approach, we increased discovery of genetic loci and identified two loci shared between neuroticism and schizophrenia and six loci shared between openness and schizophrenia. The study provides new insights into the relationship between personality traits and schizophrenia by highlighting genetic loci involved in their common genetic etiology.

## Introduction

Research on the construct and variation of human personality has revealed that personality and psychopathology are related^1–3^. This is not only the case for personality disorders but also other mental disorders such as schizophrenia^4^. However, the mechanisms underlying this relationship remain elusive. Although personality and schizophrenia may relate to each other in several ways, such as influencing the expression of one another, or predisposing for the development of one another, an intriguing question remains whether variation in personality and schizophrenia in fact share causative factors^1–3^. Both personality traits and schizophrenia are influenced by genetic factors with moderate to high heritability estimates (~0.4^5^ and 0.6–0.8^6^, respectively). Genome-wide association studies (GWAS) have revealed that the genetic architecture of personality traits and schizophrenia are composed by a number of common genetic variants with small effects^7–11^. Recently, we^10^ and others^12^ reported significant genetic correlations between several personality traits and schizophrenia^10, 12^. These findings indicate that personality traits and schizophrenia exist on a continuum in genomic space, and that some genetic variants associated with personality traits also affect susceptibility to schizophrenia^10, 12^. In order to increase the understanding of the specific molecular genetic mechanisms jointly influencing personality traits and schizophrenia, and inform the underlying biology linking these normal and pathological mental phenotypes, we here aimed to identify genetic loci shared between schizophrenia and personality traits.

The most influential personality model to emerge is the five-factor model, in which personality is defined by the five broad traits (the Big Five) neuroticism, extraversion, openness, agreeableness and conscientiousness^13, 14^. Through questionnaires, individuals are placed on continua of all five traits, which intend to account for basic behavioral tendencies that are universally present and stable throughout life^15^. Studies on personality in schizophrenia consistently find increased neuroticism, decreased extraversion and decreased conscientiousness compared to normative levels or healthy controls^16–24^, and these differences appear to persist through active and residual phases of the illness^16, 24^. Moreover, longitudinal studies report that apparently healthy individuals with high neuroticism and low extraversion were more likely to be diagnosed with schizophrenia later on^25, 26^, indicating that differences in personality might precede the onset of schizophrenia.

Despite the assembly of very large GWAS cohorts (over tens of thousands of participants) much of the genetic architecture underlying susceptibility to schizophrenia and personality traits remains to be defined, and their biological underpinnings are still largely unknown. To date, GWAS have identified hundreds of single nucleotide polymorphisms (SNPs) for schizophrenia^11^, while fewer SNPs are identified for personality traits^7–10^. Recently, we conducted a GWAS on the Big Five personality traits in the 23andMe cohort and identified six replicable variants associated with personality traits^10^. We also quantified genetic correlations between schizophrenia and personality traits using linkage disequilibrium (LD) score regression and compared personality trait GWAS data from 23andMe (n = 59,225) with GWAS data on schizophrenia from the Psychiatric Genomics Consortium (PGC; n = 17,115). We found that schizophrenia was significantly correlated with openness and conscientiousness, but not with the other personality traits^10^. However, another study reported a significant genetic correlation between neuroticism and schizophrenia using GWAS data on neuroticism in the UK Biobank sample (n = 106,716) and GWAS data on schizophrenia in the larger PGC cohort (n = 79,845)^12^. It is important to note that while correlations estimated by LD Score regression indicate the degree of shared genetic influences between two traits at the genome-wide level, they are agnostic about the individual variants jointly influencing these phenotypes.

The genome-wide significant SNPs identified by GWAS only explain a minor fraction of the estimated heritability^7–9, 11^. However, in aggregate, common SNPs explain a substantial portion of the variance of schizophrenia^27^ and personality traits^10^, indicating the presence of many variants with associations too weak to be detected using standard GWAS statistical analysis. To extract more of the “hidden heritability” within existing GWAS, we have developed Bayesian statistical tools that leverage the polygenetic overlap between complex traits^28–30^. Specifically, we condition the false discovery rate (FDR) for discovery of SNPs in a primary trait on overlapping associations in a secondary trait^28–30^. Using this methodology, we have successfully increased discovery of genetic risk loci and identified shared loci between schizophrenia and associated phenotypes including bipolar disorder^31^, cardiovascular disease risk factors^32^, and multiple sclerosis^33^. Here, we applied the same statistical approach and analyzed GWAS on the Big Five personality traits and schizophrenia to identify shared genetic loci.

## Methods

### Ethics Statement

All GWASs investigated in the current study were approved by the local ethic committees and informed consent was obtained from all participants^10, 11^. Further, the Regional Committees for Medical Research Ethics - South East Norway have evaluated the current protocol and found that no additional institutional review board approval was needed, because no individual data was used. All methods were performed in accordance with the relevant guidelines and regulations.

### Participant Samples

We obtained GWAS results in the form of summary statistic (p-values and z-scores). Data on personality traits were acquired from the 23andMe cohort (n = 59,225)^10^ and data on schizophrenia from the Psychiatric Genomics Consortium (PGC) 2 study (n = 82,315)^11^. Details of the inclusion criteria, genotyping and phenotype characteristics are described in the in the original publications^10, 11^. We corrected all p-values for inflation using a recently developed genomic inflation control procedure^31, 32, 34, 35^.

### Statistical Analyses

#### Visualizing enrichment

To visualize overlap in SNP associations, we constructed conditional Q-Q plots where we display the distribution of p-values for the primary phenotype conditional on significance levels in a secondary phenotype. Associations in the primary phenotype (schizophrenia or personality trait) were conditioned on a p-value threshold in the secondary phenotype (personality trait or schizophrenia), i.e. p < 0.1, p < 0.01 and p < 0.001. If statistical enrichment of the primary phenotype exists, there should be successive leftward deflections as levels of association with the secondary phenotype increase^28, 31–34^. The enrichment seen can be directly interpreted in terms of true discovery rate (TDR = 1 − FDR)^36^ (See Supplementary Material for details). We also constructed fold-enrichment plots, which are equivalent to conditional Q-Q plots, but provide a more direct visualization of polygenic enrichment. To assess for polygenic effects below the standard GWAS significance threshold, we focused the conditional Q-Q plots and fold-enrichment plots on SNPs with nominal log_10_(p) < 7.3 (corresponding to p > 5 × 10^−8^) and after random pruning, where one random SNP per LD block (defined by an r^2^ > 0.1) was used^11, 12, 37^. As with other methods evaluating genetic overlap using GWAS data, including LD score regression^38^ and stratified FDR^39^, complex correlations among the test-statistics may bias the estimate of the conditional FDR. The extended major histocompatibility complex (MHC)^11^ (location 25652429–33368333) and chromosomal region 8p23.1 (location 7242715–12483982) are two regions with well-known complicated LD structures^40^. Therefore we also constructed conditional Q-Q plots after excluding SNPs within these regions. Furthermore, to be conservative, we removed SNPs outside the MHC or 8p23.1 in LD (r^2^ > 0.1) with any SNP in these regions.

#### Detection of shared genetic loci using conjunction FDR

To detect shared genetic loci we used a genetic epidemiology framework based on the conjunction FDR^28^. The standard FDR framework derives from a model that assumes that the distribution of test statistics in a GWAS can be formulated as a mixture of null and non-null effects, with true associations (non-null effects) having more extreme test statistics than false associations (null effects). The FDR can be interpreted as the probability that a SNP is null given that its p-value is as small as or smaller than its observed p-value. Conjunction FDR, denoted by FDR_trait1&trait2_ is defined as the posterior probability that a SNP is null for either phenotype or both simultaneously, given that its p-values for both traits are as small as or smaller than the observed p-values^31, 32, 34^. We obtained a conservative estimate of conjunction FDR via the conditional FDR. The conditional FDR, denoted by FDR_trait1|trait2_, is defined as the posterior probability that a given SNP is null for the first trait given that the p-values for both traits are as small or smaller than the observed p-values^31–34^. A conservative estimate of FDR_trait1&trait2_ is given by the maximum between FDR_trait1|trait2_ and FDR_trait2|trait1_^41^. Hence, the conjunction FDR is the maximum of the conditional FDR for schizophrenia given a personality trait and vice versa, and SNPs that exceed a stringent conjunction FDR threshold are highly probable to be non-null in both schizophrenia and the personality trait simultaneously. While the conditional FDR can be used to reorder SNPs based on the additional information provided by the associated secondary traits, the conjunction FDR pinpoints shared loci, since a low conjunction FDR is only possible if there is an association with the two traits of interest jointly. We used an overall FDR threshold of 0.05. Given that the conjunction FDR is a genome-wide approach, it is possible that inclusion of larger LD blocks can impact the model fit and confound the results. Therefore we computed the model after random pruning and excluding SNPs within the MHC and 8p23.1, two genomic regions that show intricate LD and are associated with schizophrenia^11^ and neuroticism^10, 12^, respectively.

To visualize the location of the shared genetic variants associated with personality traits and schizophrenia, we constructed a ‘Conjunction FDR Manhattan plot’, showing all SNPs with a significant conjunction FDR within an LD block in relation to their chromosomal location. The strongest signal was identified after ranking all SNPs based on the conjunction FDR and removing SNPs in LD r^2^ > 0.1 with any higher ranked SNP. On the basis of 1KGP LD structure, significant loci identified by conjunction FDR < 0.05 were clustered into LD blocks at the LD – r^2^ > 0.1 level. These blocks are numbered (locus #) in Table 1. Any block may contain more than one SNP. Genes close to each locus were obtained from the NCBI gene database. We investigated the direction of allelic effects in the conjunctional loci by comparing the schizophrenia z-scores against the personality trait z-scores. In the Supplementary information we present ‘Conditional FDR Manhattan plots’ for schizophrenia, neuroticism and openness showing all SNPs with a significant conditional FDR within an LD block in relation to their chromosomal location. The strongest signal was identified after ranking all SNPs based on the conditional FDR and removing SNPs in LD r^2^ > 0.1 with any higher ranked SNP.

**Table 1:** Shared gene variants (conjFDR < 0.05) between SCZ and personality traits.

Locus	Marker	Nearest Gene	Chr	A1/A2	ConjFDR	Z-score SCZ	Z-score personality trait	P-value SCZ	P-value personality trait
**SCZ and Openness**									
1	rs11582132 (intergenic)	*BRINP2*	1q25.2	A/C	4.23E-02	−3.53	−3.74	4.08E-04	1.85E-04
2	rs6429422 (intronic)	*SDCCAG8*	1q43	T/G	3.58E-02	4.15	3.79	3.36E-05	1.54E-04
3	rs940404 (intronic)	*LRRC16A*	6p22.2	T/A	4.23E-02	NaN	NaN	1.61E-09	2.07E-04
4	rs3130564 (intronic)	*PSORS1C1*	6p21.33	C/T	3.58E-02	4.24	3.77	2.25E-05	1.60E-04
5	rs7779548 (3′-UTR variant)	*DGKI*	7q33	G/A	4.97E-02	4.42	3.64	9.66E-06	2.75E-04
7	rs9951150 (intergenic)	*AK093940*	18q21.2	A/G	3.58E-02	−3.76	−3.79	1.69E-04	1.49E-04
**SCZ and Neuroticism**									
6	rs2945232 (non coding transcript exon variant)	*FLJ10661*	8p23.1	T/C	1.48E-03	−4.46	5.47	8.01E-06	4.44E-08
6	rs2048656 (intergenic)	*TNKS*	8p23.1	G/A	5.80E-03	−4.19	5.01	2.84E-05	5.52E-07
8	rs11090039 (intronic)	*EP300*	22q13.2	G/A	1.18E-03	−4.49	−4.95	7.00E-06	7.46E-07

Independent complex or single gene loci (r^2^ < 0.1) with SNP(s) with a conjunction FDR (conjFDR) < 0.05 shared between SCZ and the Big Five personality traits. All significant SNPs are listed and sorted in each LD block and independent loci are listed consecutively (Locus #). All data were first corrected for genomic inflation. We also included agreeableness and conscientiousness in the conjFDR analysis, but there was no locus with conjFDR <0.05 shared between either of these traits and SCZ. Abbreviations: Chr = chromosome, A1 = allele number one, A2 = allele number two, NaN = not a number; z-score not computable due to T-A polymorphism, SCZ = schizophrenia, FDR = false discovery rate.

#### Stratified replication rate using schizophrenia substudies

We assessed whether pleiotropic enriched schizophrenia-SNPs replicate at a higher rate using the 52 schizophrenia GWAS sub-studies^11^. Sub-studies were randomly partitioned 500 times. For each random partition, half of the sub-studies were randomly assigned to the “discovery” sample and the complement to the “replication” sample. The combined discovery z-score and combined replication z-score of each SNP were calculated, and the average rate of replication (p < 0.05) was assessed across 1,000 equally spaced bins spanning the range of –log_10_(p-values) observed in the discovery samples. Cumulative replication rates were calculated independently for each of the three pleiotropic enrichment categories, as well as for all SNPs. For details, see Supplementary methods.

## Results

### Enrichment of schizophrenia SNPs due to association with personality traits, and vice versa

**Figure 1:**
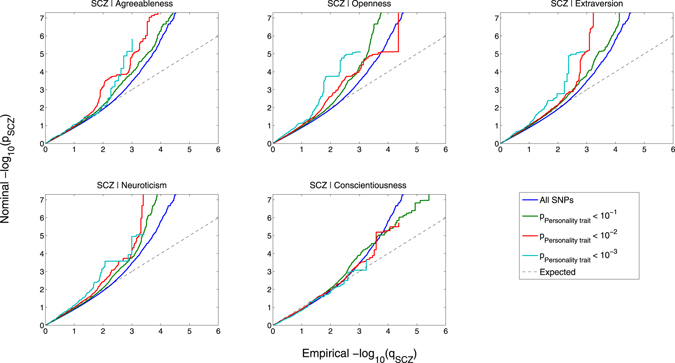
Conditional Q-Q plots of nominal versus empirical −log_10_ p-values (corrected for inflation) in schizophrenia (SCZ) below the standard GWAS threshold of p < 5 × 10^−8^ as a function of significance of association with agreeableness, openness, extraversion, neuroticism and conscientiousness at the level of −log_10_(p) ≥ 1, −log_10_(p) ≥ 2, −log_10_(p) ≥ 3 corresponding to p ≤ 0.1, p ≤ 0.01, p ≤ 0.001, respectively. Blue line indicates all SNPs. Dotted line indicates the null hypothesis.

**Figure 2:**
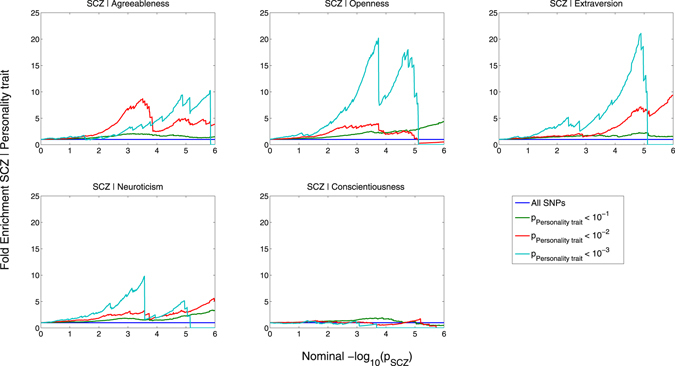
Fold-enrichment plots of enrichment versus nominal −log_10_ p-values (corrected for inflation) in schizophrenia (SCZ) below the standard GWAS threshold of p < 5 × 10^−8^ as a function of significance of association with agreeableness, openness, extraversion, neuroticism and conscientiousness at the level of −log_10_(p) ≥ 1, −log_10_(p) ≥ 2, −log_10_(p) ≥ 3 corresponding to p ≤ 0.1, p ≤ 0.01, p ≤ 0.001, respectively. Blue line indicates all SNPs.

We observed enrichment of associations with schizophrenia across different levels of association with neuroticism, agreeableness, openness and extraversion indicating polygenetic overlap between schizophrenia and these personality traits (Fig. 1). In contrast, we found no evidence for enrichment in schizophrenia conditional on conscientiousness. We also constructed the reverse conditional Q-Q plots for personality traits conditional on different levels of association with schizophrenia, demonstrating consistent polygenic enrichment in four traits, neuroticism, agreeableness, openness and extraversion, but not conscientiousness (Supplementary Figure 1). The fold-enrichment plots emphasize the polygenic enrichment in schizophrenia as a function of neuroticism, agreeableness, openness and extraversion, but not conscientiousness (Fig. 2). For progressively stringent p-value thresholds for schizophrenia SNPs [i.e., increasing values of nominal −log_10_(p)], we found approximately 10-fold enrichment using neuroticism, 20-fold enrichment using extraversion, 20-fold enrichment using openness, and 10-fold enrichment using agreeableness. In the reverse fold-enrichment plots displaying personality traits conditional on different levels of significance in schizophrenia, we found approximately 175-fold enrichment for neuroticism, 100-fold enrichment for extraversion, 10-fold enrichment for openness, and 8-fold enrichment for agreeableness, but no detectable enrichment for conscientiousness (Supplementary Figure 2). The MHC has been shown to be one of the key driving factors for enrichment of genetic association in schizophrenia^33^. Thus, we repeated the stratified Q-Q plots and fold-enrichment plots after removing all SNPs located in the MHC. As shown by the Q-Q plots and fold-enrichment plots given in Supplementary Figures 3 and 4, SNPs located within the MHC region and other SNPs in LD (r^2^ > 0.1) with such SNPs had a minor effect on enrichment. Given the strong association of chromosomal region 8p23.1 with neuroticism^10, 12^, and intricate LD in this region, we constructed conditional Q-Q plots excluding SNPs located within 8p23.1 and other SNPs in LD (r^2^ > 0.1) with such SNPs. The figures demonstrate that the genetic overlap between schizophrenia and neuroticism largely depends on associations in this region (Supplementary Figure 5).

### Susceptibility loci shared between schizophrenia and personality traits

Based on conjunction FDR < 0.05, we identified six loci shared between openness and schizophrenia annotated to genes *BRINP2* (rs11582132, intergenic variant), *SDCCAG8* (rs6429422, intronic), *LRRC16A* (rs940404, intronic), *PSORS1C1* (rs3130564, intronic), *DGKI* (3′-UTR variant), and *AK093940* (rs9951150, intergenic) (Table 1). *PSORS1C1* maps onto the MHC region. Due to the intricate LD in this region, we consider this conjunctional hit as to reflect the involvement of MHC in both schizophrenia and openness rather than *PSORS1C1* specifically. Further, we identified three loci shared between neuroticism and schizophrenia, which were annotated to *FLJ10661* (rs2945232; non-coding transcript exon variant), *TNKS* (rs2048656; intergenic) and *EP300* (rs11090039; intronic) (Table 1). *FLJ10661* and *TNKS* map onto chromosomal band 8p23.1, a region spanning ~4 Mb with extended LD containing at least 36 genes^10^. Consequently, we consider these hits as to reflect the involvement of 8p23.1 in schizophrenia and neuroticism rather than these specific variants.

**Figure 3:**
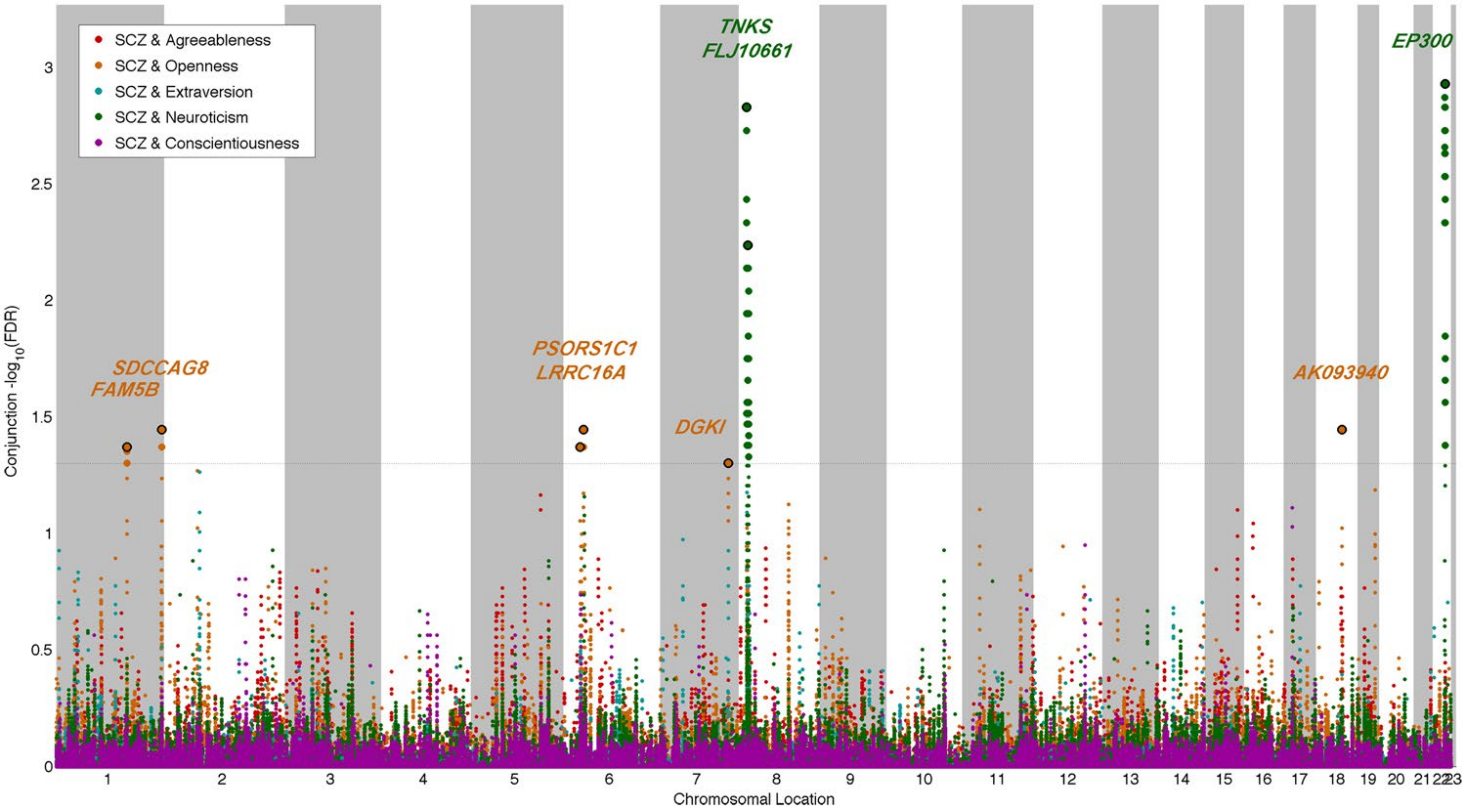
‘Conjunction FDR Manhattan plot’ of conjunction (FDR < 0.05) values for schizophrenia (SCZ) and agreeableness, openness, extraversion, neuroticism and conscientiousness. SNPs with conjunction FDR < 0.05 (i.e., −log_10_ FDR > 1.3) are shown with enlarged data points. A black circle around the enlarged data points indicates the most significant SNP in each LD block and this SNP was annotated with the closest gene which is listed above the symbols in each locus. The figure shows the localization of the ‘conjunctional loci’, and further details are provided in Table 1.

To visualize the shared loci, we constructed a conjunction FDR Manhattan plot (Fig. 3). All SNPs without pruning are shown, and the strongest signal in each LD block is encircled in black. The enlarged data points represent the significant SNPs (FDR_trait1&trait2_ < 0.05), whereas the small points represent the non-significant SNPs. We also constructed conditional FDR Manhattan plots for neuroticism given schizophrenia, openness given schizophrenia, and schizophrenia given neuroticism and openness, to visualize conditional loci (Supplementary Figures 6–8, respectively). Next, we evaluated the directionality of allelic effects in the conjunctional loci comparing z-scores in schizophrenia against z-scores in the personality trait (Table 1). For five loci shared between openness and schizophrenia (*BRINP2*, *SDCCAG8*, *PSORS1C1*, *DGKI* and *AK093940*), the effect directions were concordant. Due to a T-A polymorphism, z-scores were not computable for the locus at *LRRC16A* (rs940404). The 8p23.1 loci show inverse associations in neuroticism and schizophrenia, while the *EP300* locus shows concordant associations.

### Replication rates in schizophrenia are increased by personality trait association

To address the possibility that the observed pattern of differential enrichment results from spurious (i.e., non-generalizable) associations, we also calculated the empirical replication rate across the independent substudies contributing to the schizophrenia GWAS meta-analysis^11^. Supplementary Figures 9 and 10 show the empirical cumulative replication rate plots as a function of nominal p-value, the same categories as for the conditional Q-Q and fold-enrichment plots in Figs 1 and 2. We show that replication rates in schizophrenia SNPs are increased by conditioning on increasing levels of association with openness and neuroticism although this is not evident for the category −log_10_(p_neuroticism_) ≥ 3). Consistent with the pattern observed for replication rates in schizophrenia substudies, we found that the effect sizes of SNPs in enriched categories (for example, −log_10_(p_openness_) ≥ 3) replicated better than effect sizes of SNPs in less-enriched categories (for example, −log_10_(p_openness_) ≥ 1; Supplementary Figures 10 and 11). This indicates that the fidelity of replication effect sizes is closely related to the conditional TDR.

## Discussion

In the present study, we analyzed GWAS data on schizophrenia and the Big Five personality traits using the conditional FDR approach to evaluate overlap in common genetic variants. By conditioning on overlapping SNP associations, we were able to identify six loci shared between openness and schizophrenia, and two loci shared between neuroticism and schizophrenia. To validate our approach, we show that schizophrenia SNPs replicate at a higher rate across independent schizophrenia sub-studies as a function of association with personality traits. Altogether, our study provides new insights into the genetic architecture of schizophrenia and personality traits by identifying genetic loci that link personality trait variation and susceptibility to schizophrenia. The findings comply with accumulating evidence from genetics and neuroscience suggesting that mental disorders are not discontinuous with normal variation in neurobiological and behavior dimensions^42, 43^.

Recently, we estimated a significant positive genetic correlation between schizophrenia and openness using LD score regression^10^. In line with this result, five of the loci here found to be shared between schizophrenia and openness showed the same direction of allelic effects in the phenotypes, while one locus showed ambiguous effect directions (rs940404; *LRRC16A*) (Table 1). To our knowledge, none of the loci shared between openness and schizophrenia are identified in prior genetic studies on personality, whereas all loci are implicated in schizophrenia^11, 44, 45^. Levels of openness, which captures intellectual curiosity, unconventional thinking, imagination and versatility^13, 46^, appear to not differ from normality among patients with schizophrenia^16–24^. Nevertheless, it is worth noting that both openness and schizophrenia are linked to heightened dopamine activity^47, 48^, and are positively correlated with measures of creativity^49–52^, which could relate to the positive correlation among genetic effects shared between schizophrenia and openness. To determine the actual neurobiological and behavioral implications of the identified genetic variants and their role in openness and schizophrenia further studies are needed.

Schizophrenia is associated with high levels of neuroticism^16–24^, which can be defined as the tendency to experience negative emotions such as anxiety, anger and depression in stressful situations^46^. While one recent study estimated a significant positive genetic correlation between schizophrenia and neuroticism^12^, an analysis of different GWAS data did not reveal a significant genetic correlation between these phenotypes^10^. Here, we supplement these findings by identifying two genetic loci shared between schizophrenia and neuroticism. The intronic locus within *EP300* (rs11090039) shows the same direction of allelic effects in schizophrenia and neuroticism, while the effect directions were opposite at chromosomal region 8p23.1 (Table 1). Notably, the *EP300* locus was genome-wide significant in both the primary schizophrenia GWAS^11^ and the 23andMe GWAS on neuroticism^10^. No loci within 8p23.1 reached genome-wide significance in the schizophrenia GWAS^11^. However, the gene *TNKS* is twice implicated as a gene affected by *de novo* mutations in schizophrenia^53, 54^. Recently, two independent GWAS on neuroticism reported the strongest signal of association within 8p23.1, clearly demonstrating the importance of this locus in neuroticism^10, 12^. The opposite effect directions in schizophrenia and neuroticism detected at 8p23.1 could be attributable to different haplotypes/gene alleles involved in the phenotypes in this region^55^, or indicate that the same haplotypes/gene alleles are involved in both schizophrenia and neuroticism but the underlying biological mechanisms are distinct^55^. Fine-mapping studies are required to pinpoint the causative variants in 8p23.1. Of interest, the distal 15 Mb of the 8p chromosomal region (including 8p23.1) has been implicated as a potential hub for neuropsychiatric disorders^56^. The region is subject to structural variants associated with schizophrenia and autism, and shows high linkage to schizophrenia, bipolar disorder, and neuroticism among other phenotypes (for review see ref. 56).

Despite the clinical association between schizophrenia and low levels of conscientiousness^16–24^, and a significant negative genetic correlation between schizophrenia and conscientiousness^10^, we did not identify any gene loci shared between these phenotypes using the conditional FDR. The discrepancy may result from the different SCZ GWAS data on schizophrenia analyzed in these studies. In the LD score regression analysis, data from a smaller schizophrenia GWAS cohort (n = 17,115)^57^ were analyzed^10^, while the present study analyzed GWAS data on schizophrenia in the recent and larger PGC2 cohort (n = 82,315)^11^. Another possibility is that the observed negative genetic correlation between conscientiousness and schizophrenia using LD score regression is driven by a number of loci with too small effects to be detected by the conditional FDR approach. Further, although schizophrenia is associated with decreased levels of extraversion^16–24^, we did not detect any significant loci shared between schizophrenia and extraversion in the present study. However, the pleiotropic enrichment demonstrated by the conditional Q-Q plots (Figure 1) indicates overlapping SNP associations between schizophrenia and extraversion. These findings may suggest that the current GWAS sample sizes for extraversion are not sufficiently powered to detect any shared genetic variants with schizophrenia^10^.

The conditional FDR methodology has been useful to elucidate genetic overlap in several complex phenotypes including neuropsychiatric disorders^31, 33^, cardiovascular disease traits^32^ and immune-related diseases^34^. The current findings in the Big Five personality traits demonstrate that it may also be applied to genetic research on personality. By combining the 23andMe personality sample with the independent PGC2 schizophrenia GWAS^11^, we identified significant overlapping signals in a total of eight independent loci. Apart from the loci within chromosomal region 8p23.1, all conjunctional loci were identified by the schizophrenia GWAS^11^. However, in the 23andMe cohort, only loci associated with neuroticism were identified^10^. This illustrates the increased power of our combined analytical approach. Although it is likely that the shared loci discovered here would have been identified if the sample sizes in original GWAS had been adequately large, we show how combining summary statistics from independent samples provides an asset for gene discovery without the extra cost and resources needed to obtain new samples. Another strength of our conditional FDR approach is that it enables detection of overlapping variants even when the direction of effect is inconsistent across loci.

In conclusion, we provide new insights into the genetic etiology underlying schizophrenia and personality traits by increasing discovery of genetic loci and identifying common genetic variants shared between schizophrenia and the Big Five personality traits openness and neuroticism. By highlighting genetic loci that transcend boundaries between schizophrenia and personality dimensions, our study may align with novel conceptual approaches to psychiatric nosologies, in which mental disorders are considered continuous with normal variation in psychological and neural phenotypes^42, 43^. Further investigation is required to determine the biological implications of the identified genetic variants to elucidate how neurobiological processes are altered to influence personality and risk of schizophrenia.

## Electronic supplementary material

Supplementary Information
