# Supplementary material for: Identification of genetic loci shared between schizophrenia and the Big Five personality traits
Source: Sci Rep. 2017 May 22;7:2222. doi: 10.1038/s41598-017-02346-3 (PMC5440373; doi:10.1038/s41598-017-02346-3)
Supplement: Supplementary file 1 — Supplementary Information [file 41598_2017_2346_MOESM1_ESM.pdf]

SUPPLEMENTARY INFORMATION

Manuscript title:

Identification of genetic loci shared between schizophrenia and the Big Five personality traits

Author list:

Olav B Smeland (M.D. Ph.D.)[1], Yunpeng Wang (Ph.D.)[1,2], Min-Tzu Lo (Ph.D.)[3], Wen Li (Ph.D.)[1], Oleksandr Frei (Ph.D.)[1], Aree Witoelar (Ph.D.)[1], Martin Tesli (M.D. Ph.D.)[1,4], David A Hinds (Ph.D.)[5], Joyce Y Tung (Ph.D.)[5], Srdjan Djurovic (Ph.D.)[6,7], Chi-Hua Chen (Ph.D.)[3], Anders M Dale (Ph.D.)[2,3,8], Ole A Andreassen (M.D. Ph.D.)[1]

[1]NORMENT, KG Jebsen Centre for Psychosis Research, Institute of Clinical Medicine, University of Oslo and Division of Mental Health and Addiction, Oslo University Hospital, 0407 Oslo, Norway; [2]Department of Neurosciences, University of California San Diego, La Jolla, CA 92093, United States of America; [3]Department of Radiology, University of California, San Diego, La Jolla, CA 92093, United States of America; [4]Lovisenberg Diakonale Hospital, 0456 Oslo, Norway; [5]23andMe, Inc. Mountain View, CA 9404 United States of America; [8]Department of Medical Genetics, Oslo University Hospital, Oslo, Norway; [7]NORMENT, KG Jebsen Centre for Psychosis Research, Department of Clinical Science, University of Bergen, Bergen, Norway; [8]Department of Psychiatry, University of California, San Diego, La Jolla, CA, USA

SUPPLEMENTARY METHODS

Conditional True Discovery Rate (TDR)

The 'enrichment' seen in the conditional Q-Q plots and fold-enrichment plots can be directly interpreted in terms of true discovery rate (TDR = 1 – FDR)[1]. More specifically, for a given p-value cutoff, the FDR is defined as

$$\mathrm{FDR}(p) = \pi_0 F_0(p) / F(p), \qquad [1]$$

where π_0 is the proportion of null SNPs, F_0 is the null cumulative distribution function (cdf), and F is the cdf of all SNPs, both null and non-null[2]. Under the null hypothesis, F_0 is the cdf of the uniform distribution on the unit interval [0,1], so that Eq. [1] reduces to

$$\mathrm{FDR}(p) = \pi_0\, p / F(p), \qquad [2]$$

The cdf F can be estimated by the empirical cdf $q = N_p / N$, where N_p is the number of SNPs with p-values less than or equal to p, and N is the total number of SNPs. Replacing F by q in Eq. [2], we get

$$\text{Estimated FDR}(p) = \pi_0\, p / q, \qquad [3]$$

which is biased upwards as an estimate of the FDR[3]. Replacing π in Equation [3] with unity gives an estimated FDR that is further biased upward;

$$q^* = p/q \qquad [4]$$

If π_0 is close to one, as is likely true for most GWASs, the increase in bias from Eq. [3] is minimal. The quantity 1 – p/q, is therefore biased downward, and hence a conservative estimate of the TDR. Referring to the Q-Q plots, we see that q* is equivalent to the nominal p-value divided by the empirical quantile, as defined earlier. We can thus read the FDR estimate directly

off the Q-Q plot as

$$-\log_{10}(q^*) = \log_{10}(q) - \log_{10}(p), \qquad [5]$$

i.e. the horizontal shift of the curves in the Q-Q plots from the expected line x = y, with a larger shift corresponding to a smaller FDR. This is illustrated in Figure 1 and Supplementary Figure 1.

Conditional Q-Q plots

Q-Q plots compare a nominal probability distribution against an empirical distribution. In the presence of all null relationships, nominal p-values form a straight line on a Q-Q plot when plotted against the empirical distribution. For SCZ, neuroticism, extraversion, openness agreeableness, and conscientiousness SNPs and for each categorical subset (strata), $-\log_{10}$ nominal p-values were plotted against $-\log_{10}$ empirical p-values (conditional Q-Q plots). Leftward deflections of the observed distribution from the projected null line reflect increased tail probabilities in the distribution of test statistics (z-scores) and consequently an over-abundance of low p-values compared to that expected by chance, also named ‘enrichment’.

Under large-scale testing paradigms, such as GWAS, quantitative estimates of likely true associations can be estimated from the distributions of summary statistics[2,4]. One common method for visualizing the enrichment of statistical association relative to that expected under the global null hypothesis is through Q-Q plots of nominal p-values obtained from GWAS summary statistics. The usual Q-Q curve has as the y-ordinate the nominal p-value, denoted by “p”, and as the x-ordinate the corresponding value of the empirical cdf, denoted by “q”. Under the global null hypothesis the theoretical distribution is uniform on the interval [0,1]. As is common in GWAS, we instead plot $-\log_{10}$ p against $-\log_{10}$ q to emphasize tail probabilities of the theoretical and empirical distributions. Therefore, genetic enrichment results in a leftward shift in the Q-Q curve,

corresponding to a larger fraction of SNPs with nominal $-\log_{10}$ p-value greater than or equal to a given threshold. Conditional Q-Q plots are constructed by creating subsets of SNPs based on levels of an auxiliary measure for each SNP, and computing Q-Q plots separately for each level. If SNP enrichment is captured by variation in the auxiliary measure, this is expressed as successive leftward deflections in a conditional Q-Q plot as levels of the auxiliary measure increase. We constructed conditional Q-Q plots of empirical quantiles of nominal $-\log_{10}$ values for SNP association with SCZ for all SNPs, and for subsets (strata) of SNPs determined by the nominal p-values of their association with each personality trait, and vice versa. Specifically, we computed the empirical cumulative distribution of nominal p-values for a given phenotype for all SNPs and for SNPs with significance levels below the indicated cut-offs for the other phenotypes ($-\log_{10}(p) \geq 1$, $-\log_{10}(p) \geq 2$, $-\log_{10}(p) \geq 3$ corresponding to $p < 0.1$, $p < 0.01$, $p < 0.001$ respectively). The nominal p-values ($-\log_{10}(p)$) are plotted on the y-axis, and the empirical quantiles ($-\log_{10}(q)$, where $q=1-cdf(p)$) are plotted on the x-axis. To assess for polygenic effects below the standard GWAS significance threshold, we focused the conditional Q-Q plots on SNPs with nominal $-\log_{10}(p) < 7.3$ (corresponding to $p > 5x10^{-8}$).

Fold enrichment plots

To assess genetic enrichment, we also constructed fold enrichment plots conditional by 'pleiotropic' effects. For a given associated phenotype, enrichment for pleiotropic signals is present if the degree of deflection from the expected null line is dependent on SNP associations with the second phenotype. We constructed fold-enrichment plots of empirical quantiles of nominal $-\log_{10}$ values for SNP association with SCZ for all SNPs, and for subsets (strata) of SNPs determined by the nominal p-values of their association with each personality trait, and vice versa. Specifically, we computed the empirical cumulative distribution of nominal p-values for a

given phenotype for all SNPs and for SNPs with significance levels below the indicated cut-offs for the other phenotypes ($-\log_{10}(p) \geq 1$, $-\log_{10}(p) \geq 2$, $-\log_{10}(p) \geq 3$ corresponding to $p < 0.1$, $p < 0.01$, $p < 0.001$ respectively). The nominal p-values ($-\log_{10}(p)$) are plotted on the x-axis, and fold enrichment in the first phenotype as a function of the second phenotype is plotted on the x-axis. To assess for polygenic effects below the standard GWAS significance threshold, we focused the fold enrichment plots on SNPs with nominal $-\log10(p) < 7.3$ (corresponding to $p > 5x10^{-8}$).

Stratified replication rate using PGC2 schizophrenia substudies. Replication rate was assessed using schizophrenia GWAS data[5]. The 52 PGC schizophrenia sub-studies were randomly partitioned 500 times. For each random partition, half of the sub-studies were randomly assigned to the "discovery" sample and the complement to the "replication" sample. We calculated the combined discovery z-score and the combined replication z-score of each SNP by calculating the meta-analysis z-score using a weighted sum, with weights proportional to the square root of the sample size of each study[6]. For discovery samples, the z-scores were converted to two-tailed p-values. For replication samples, z-scores were converted to one-tailed p-values, preserving the direction of effect in the discovery sample. For each of the 500 discovery-replication pairs, cumulative rates of replication were calculated over 1000 equally spaced bins spanning the range of $-\log_{10}$(p-values) observed in the discovery samples. The cumulative replication rate for any bin was calculated as the proportion of SNPs with a $-\log_{10}$(discovery p-value) greater than the lower bound of the bin with a replication p-value <0.05. Cumulative replication rates were calculated independently for each of the three pleiotropic enrichment categories, as well as for all SNPs. For each category, the cumulative replication rate for each bin was averaged across the 500 discovery-replication pairs. The vertical intercept is the overall replication rate. We

constructed replication plots after random pruning, where one random SNP per LD block (defined by an $r^2 > 0.1$) was used, and excluded SNPs within the MHC-region and other SNPs in LD with such SNPs.

Conditional replication effect size. We directly evaluated the relationship of replication effect size of the discovery samples versus replication samples for each SNP using the same sampling method used for estimating cumulative replication rates (see above) across 100 equally spaced bins spanning the range of z-scores observed in the discovery samples. The effect sizes were conditioned on pleiotropic enrichment categories.

References for Supplementary Information

1 Benjamini, Y. & Hochberg, Y. in *Journal of the Royal Statistical Society. Series B (Methodological)* Vol. 57 289-300 (Blackwell Publishing, 1995).

2 Efron, B. Size, power and false discovery rates. *The Annals of Statistics* **35**, 1351–1377 (2007).

3 Purcell, S. *et al.* PLINK: a tool set for whole-genome association and population-based linkage analyses. *American journal of human genetics* **81**, 559-575, doi:10.1086/519795 (2007).

4 Schweder, T. & Spjotvoll, E. Plots of P-Values to Evaluate Many Tests Simultaneously. *Biometrika* **69**, 493-502 (1982).

5 Schizophrenia Working Group of the Psychiatric Genomics, C. Biological insights from 108 schizophrenia-associated genetic loci. *Nature* **511**, 421-427, doi:10.1038/nature13595 (2014).

6 Stouffer, S. A., Suchman, E. A., DeVinney, L. C., Star, S. A. & Williams, R. M. J. *The American soldier: Adjustment during Army Life*. (Princeton University Press, 1949).

SUPPLEMENTARY FIGURES

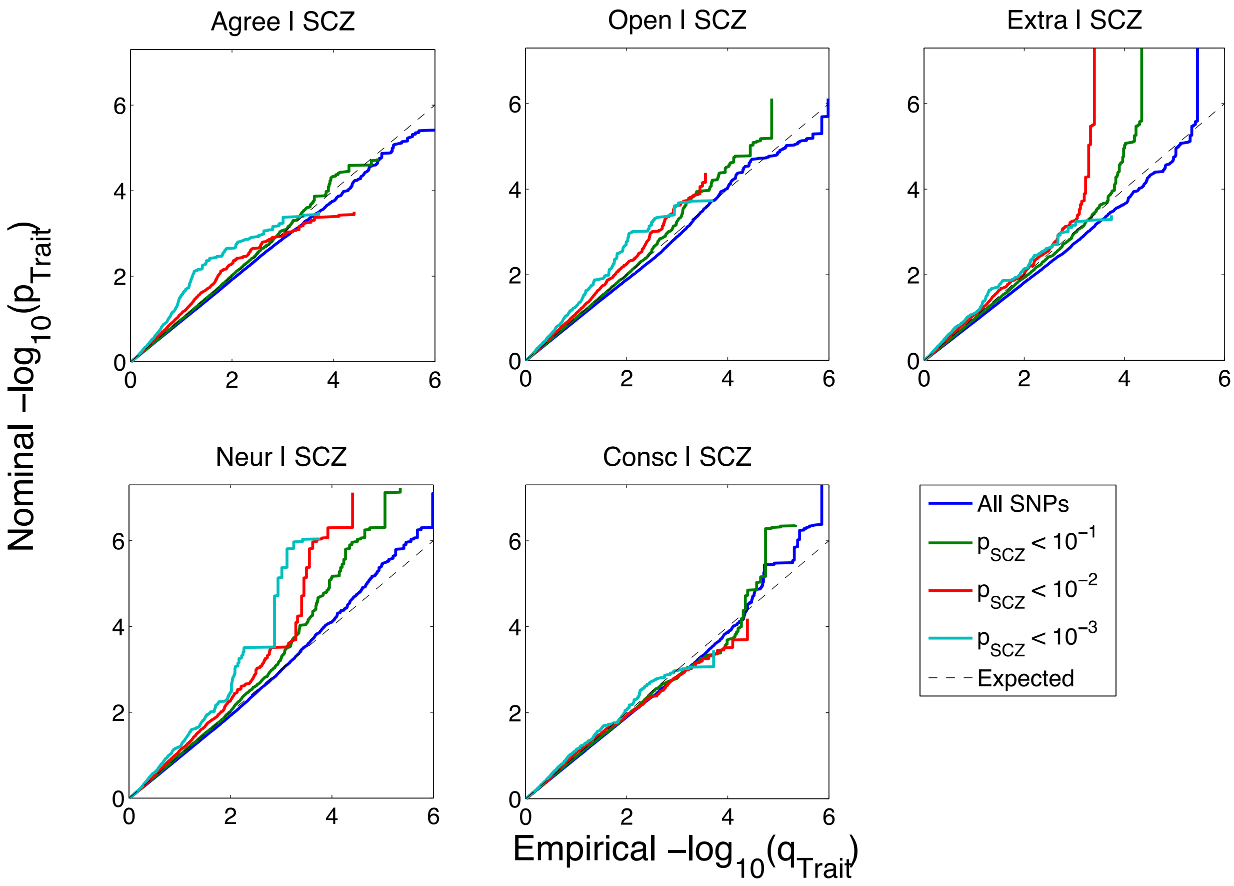

Supplementary Figure 1.

Conditional Q-Q plots of nominal versus empirical $-\log_{10}$ p-values (corrected for inflation) in agreeableness (AGREE), openness (OPEN) extraversion (EXTRA), neuroticism (NEUR) and conscientiousness (CONSC) below the standard GWAS threshold of $p<5\times10^{-8}$ as a function of significance of association with schizophrenia (SCZ) at the level of $-\log_{10}(p)\geqslant1$, $-\log_{10}(p)\geqslant2$, $-\log_{10}(p)\geqslant3$ corresponding to $p\leqslant0.1$, $p\leqslant0.01$, $p\leqslant0.001$, respectively. Blue line indicates all SNPs. Dotted line indicates the null hypothesis.

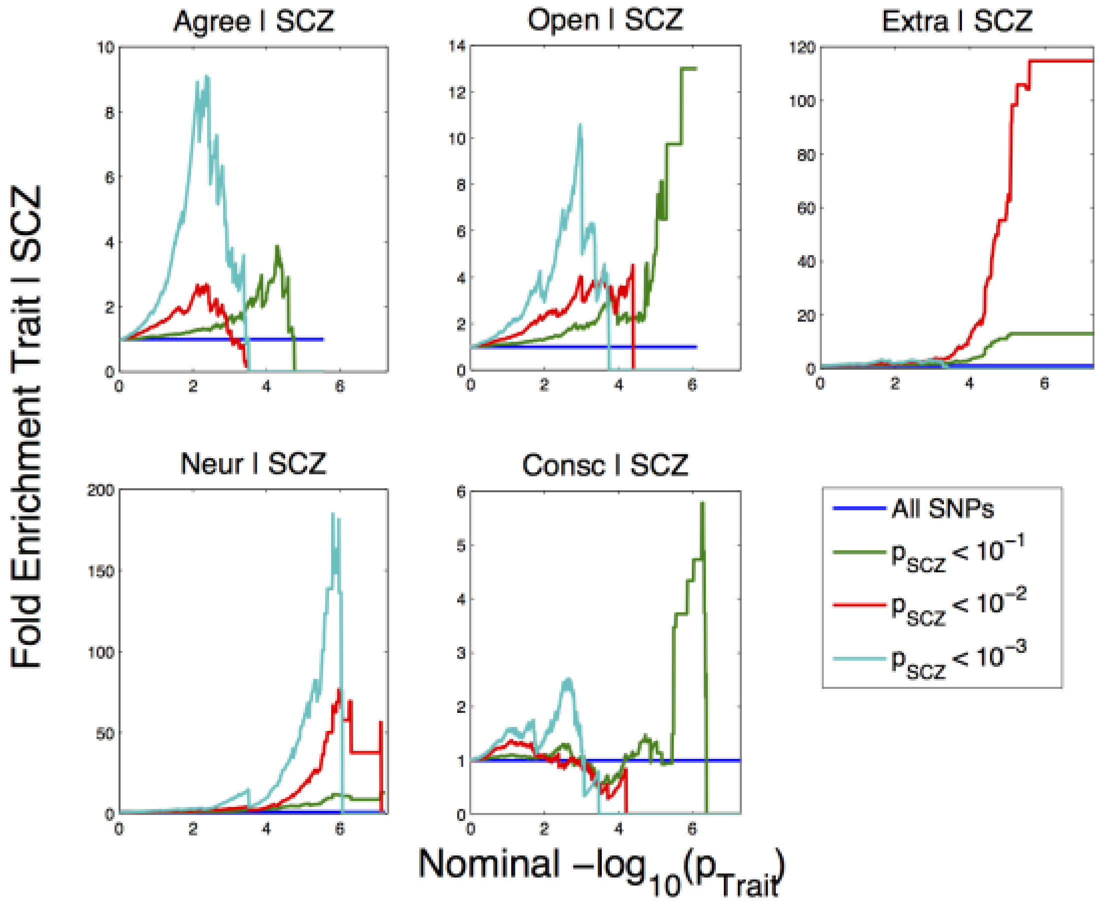

Supplementary Figure 2. Fold-enrichment plots of enrichment versus nominal $-\log_{10}$ p-values (corrected for inflation) in agreeableness (AGREE), openness (OPEN) extraversion (EXTRA), neuroticism (NEUR) and conscientiousness (CONSC) below the standard GWAS threshold of $p < 5\times10^{-8}$ as a function of significance of association with schizophrenia (SCZ) at the level of $-\log_{10}(p)\geqslant1$, $-\log_{10}(p)\geqslant2$, $-\log_{10}(p)\geqslant3$ corresponding to $p\leqslant0.1$, $p\leqslant0.01$, $p\leqslant0.001$, respectively. Blue line indicates all SNPs.

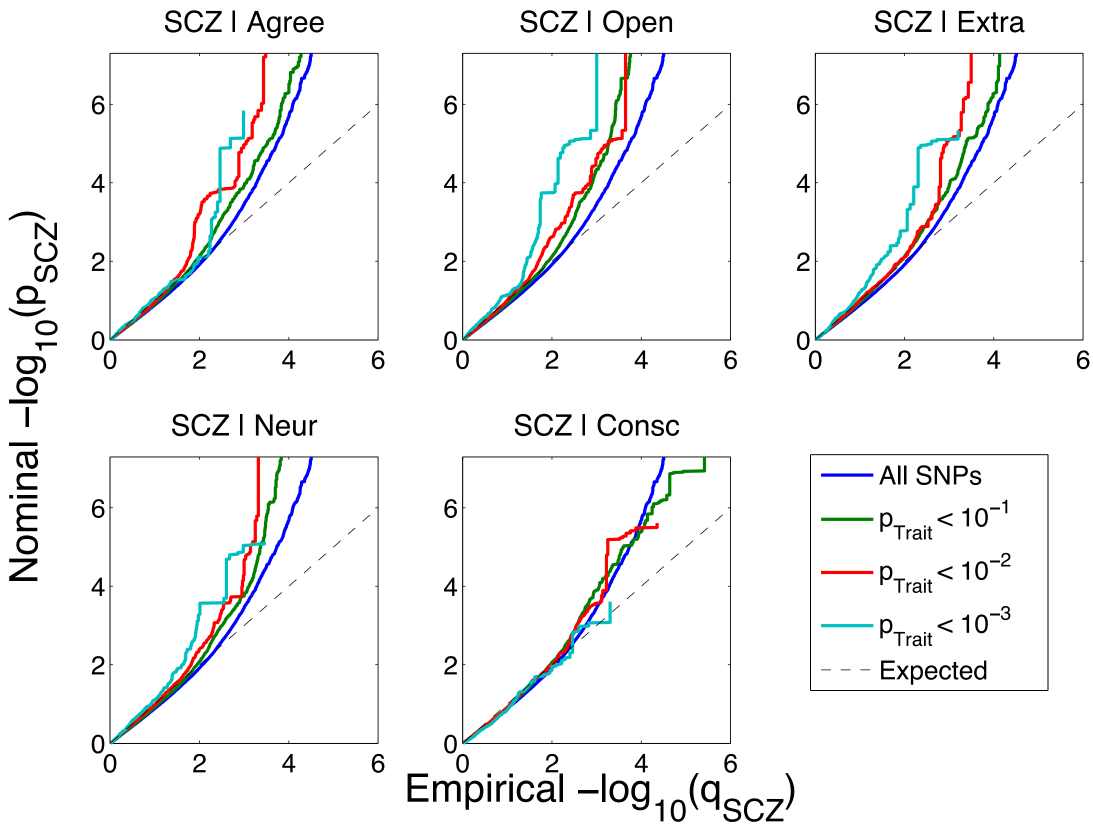

Supplementary Figure 3. Conditional Q-Q plots of nominal versus empirical $-\log_{10}$ p-values (corrected for inflation) in schizophrenia (SCZ) below the standard GWAS threshold of $p<5\times10^{-8}$ as a function of significance of association with agreeableness (AGREE), openness (OPEN) extraversion (EXTRA), neuroticism (NEUR) and conscientiousness (CONSC) at the level of $-\log_{10}(p)\geqslant1$, $-\log_{10}(p)\geqslant2$, $-\log_{10}(p)\geqslant3$ corresponding to $p\leqslant0.1$, $p\leqslant0.01$, $p\leqslant0.001$, respectively, after removing SNPs located within the MHC region and other SNPs in LD ($r^2 > 0.1$) with such SNPs. Blue line indicates all SNPs. Dotted line indicates the null hypothesis.

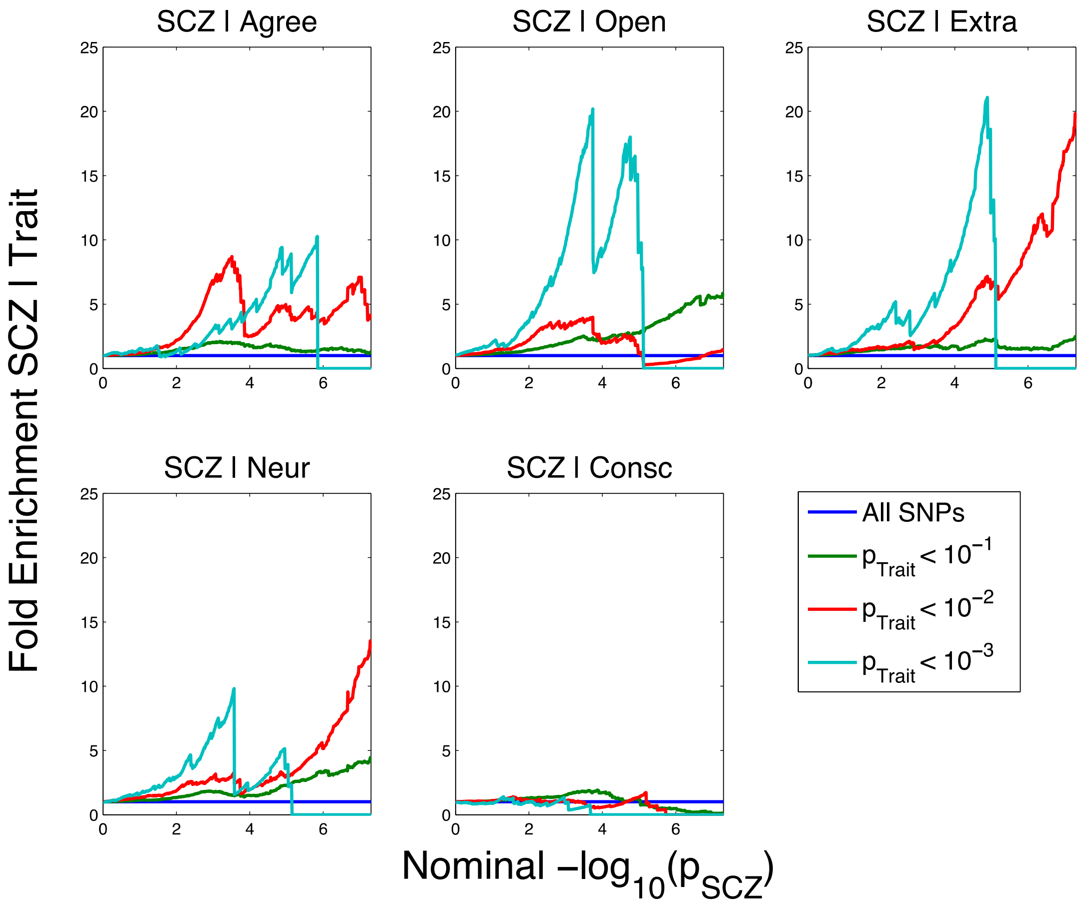

Supplementary Figure 4. Fold-enrichment plots of enrichment versus nominal $-\log_{10}$ p-values (corrected for inflation) in schizophrenia (SCZ) below the standard GWAS threshold of $p<5\times10^{-8}$ as a function of significance of association with agreeableness (AGREE), openness (OPEN) extraversion (EXTRA), neuroticism (NEUR) and conscientiousness (CONSC) at the level of $-\log_{10}(p)\geqslant1$, $-\log_{10}(p)\geqslant2$, $-\log_{10}(p)\geqslant3$ corresponding to $p\leqslant0.1$, $p\leqslant0.01$, $p\leqslant0.001$, respectively, after removing SNPs located within the MHC region and other SNPs in LD ($r^2 > 0.1$) with such SNPs. Blue line indicates all SNPs.

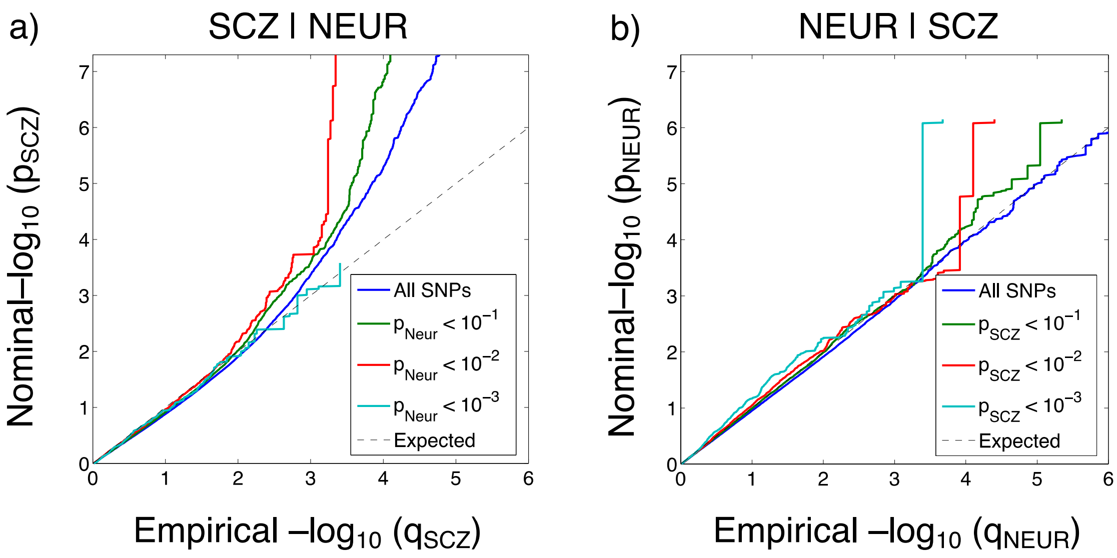

Supplementary Figure 5. Conditional Q-Q plots of nominal versus empirical $-\log_{10}$ p-values (corrected for inflation) in (a) schizophrenia (SCZ) as a function of significance of association with neuroticism (NEUR) and (b) NEUR as a function of significance of association with SCZ at the level of $-\log_{10}(p)\geqslant1$, $-\log_{10}(p)\geqslant2$, $-\log_{10}(p)\geqslant3$ corresponding to $p\leqslant0.1$, $p\leqslant0.01$, $p\leqslant0.001$, respectively, after removing SNPs located within the MHC region and chromosomal region 8p23.1 and other SNPs in LD ($r^2 > 0.1$) with such SNPs. Blue line indicates all SNPs. Dotted line indicates the null hypothesis.

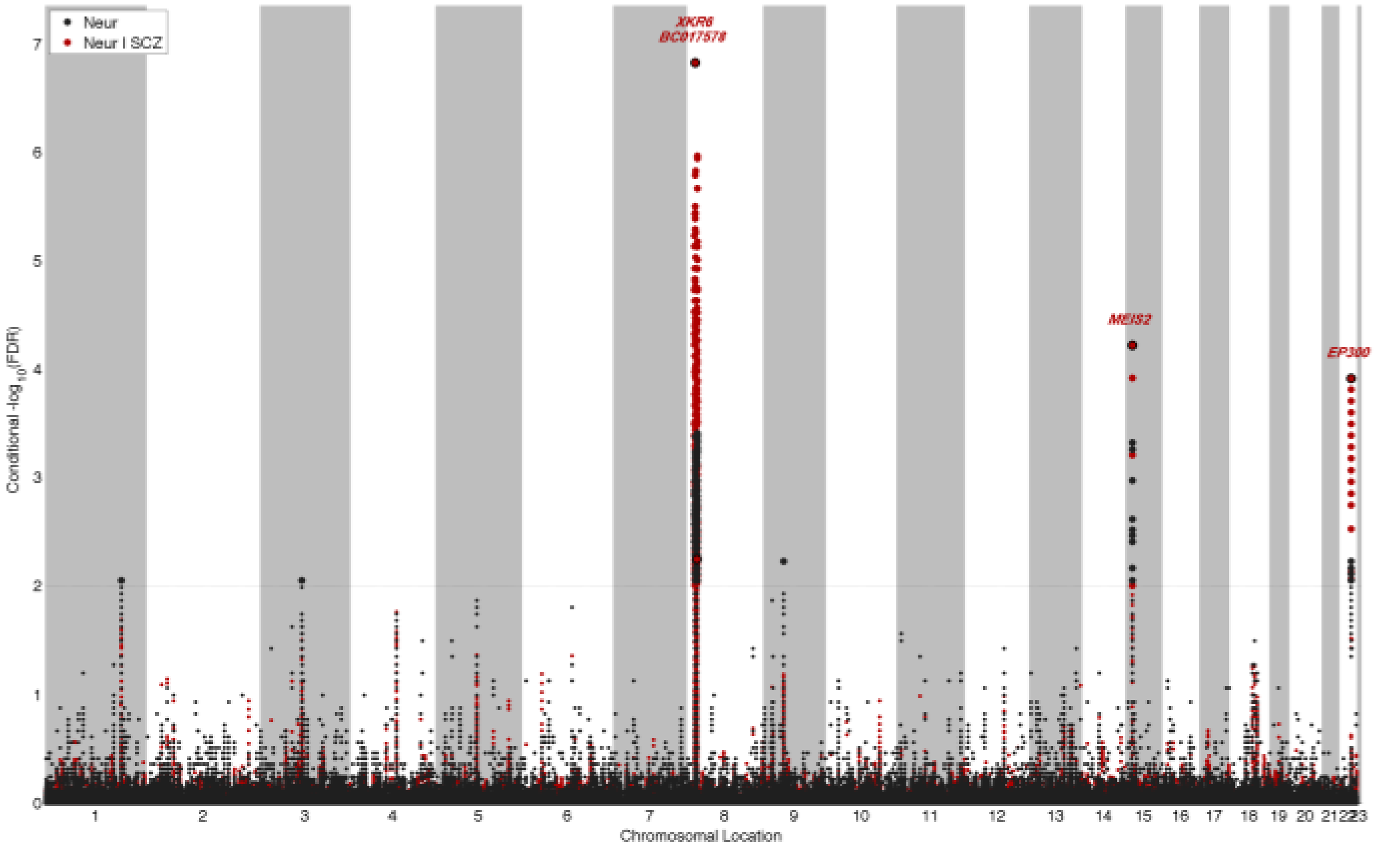

Supplementary Figure 6. ‘Conditional FDR Manhattan plot’. Conditional FDR Manhattan plot of conditional –$\log_{10}$ (FDR) values for neuroticism (NEUR) alone (black) and NEUR conditioned on schizophrenia (SCZ; NEUR|SCZ, red). SNPs with conditional –$\log_{10}$ FDR> FDR>2.0 (that is, FDR<0.01) are shown with large points. A black line around the large points indicates the most significant SNP in each LD block. This SNP is annotated with the closest gene.

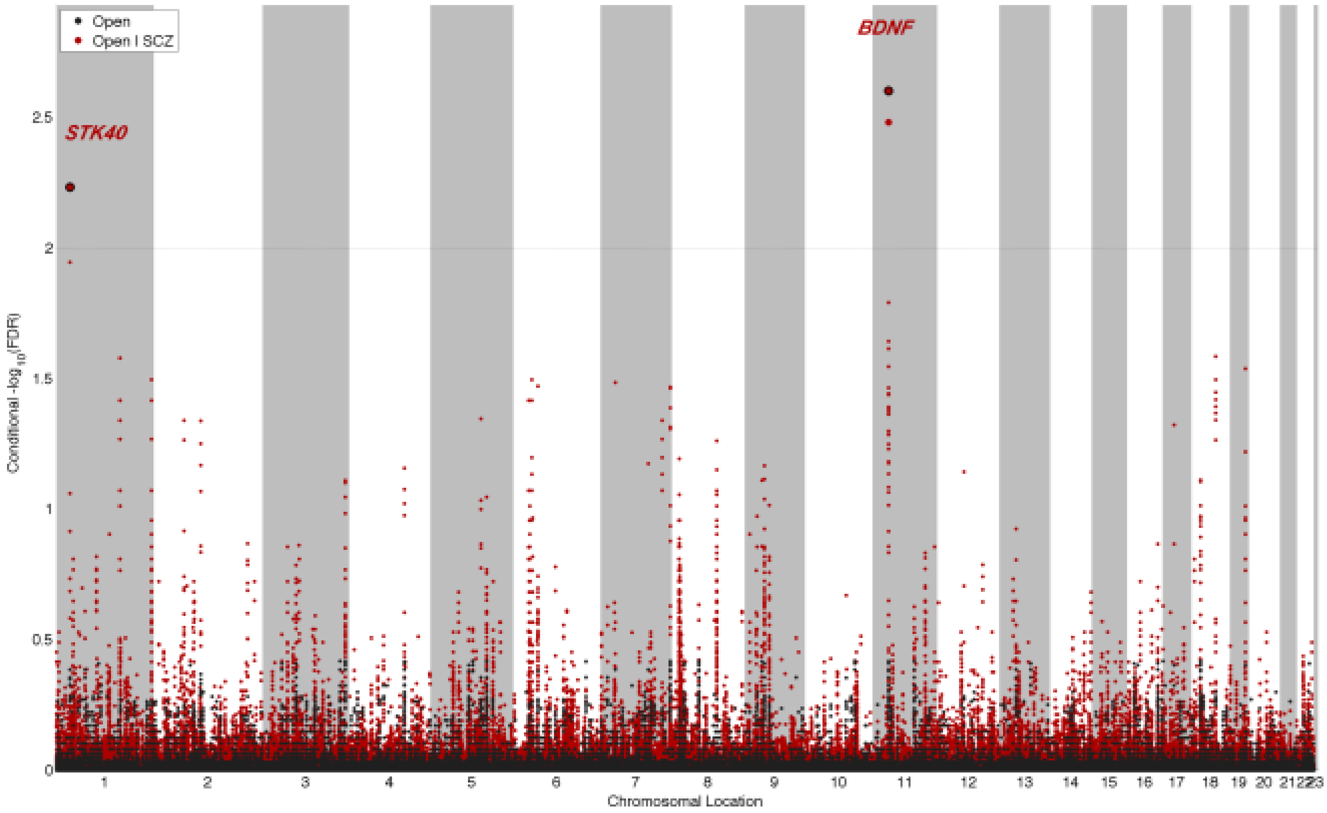

Supplementary Figure 7. 'Conditional FDR Manhattan plot'. Conditional FDR Manhattan plot of conditional $-\log_{10}$ (FDR) values for openness (OPEN) alone (black) and OPEN conditioned on schizophrenia (SCZ; OPEN|SCZ, red). SNPs with conditional $-\log_{10}$ FDR>2.0 (that is, FDR<0.01) are shown with large points. A black line around the large points indicates the most significant SNP in each LD block. This SNP is annotated with the closest gene.

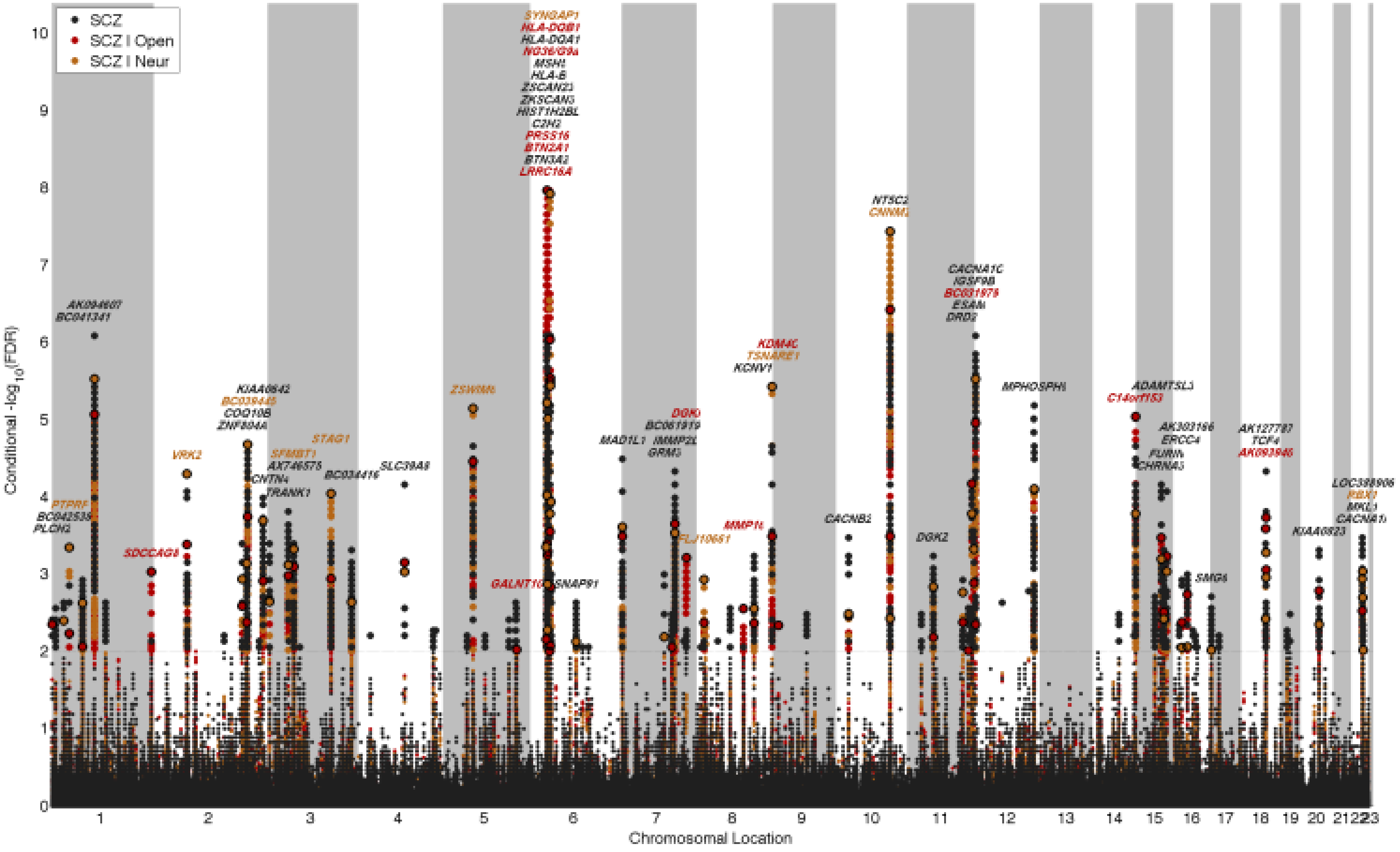

Supplementary figure 8. 'Conditional FDR Manhattan plot'. Conditional FDR Manhattan plot of conditional $-\log_{10}$ (FDR) values for schizophrenia (SCZ) alone (black) and SCZ conditioned on openness (OPEN; SCZ|OPEN, red) and neuroticism (NEUR; SCZ|NEUR, orange). SNPs with conditional $-\log_{10}$ FDR >2.0 (that is, FDR <0.01) are shown with large points. A black line around the large points indicates the most significant SNP in each LD block. This SNP is annotated with the closest gene.

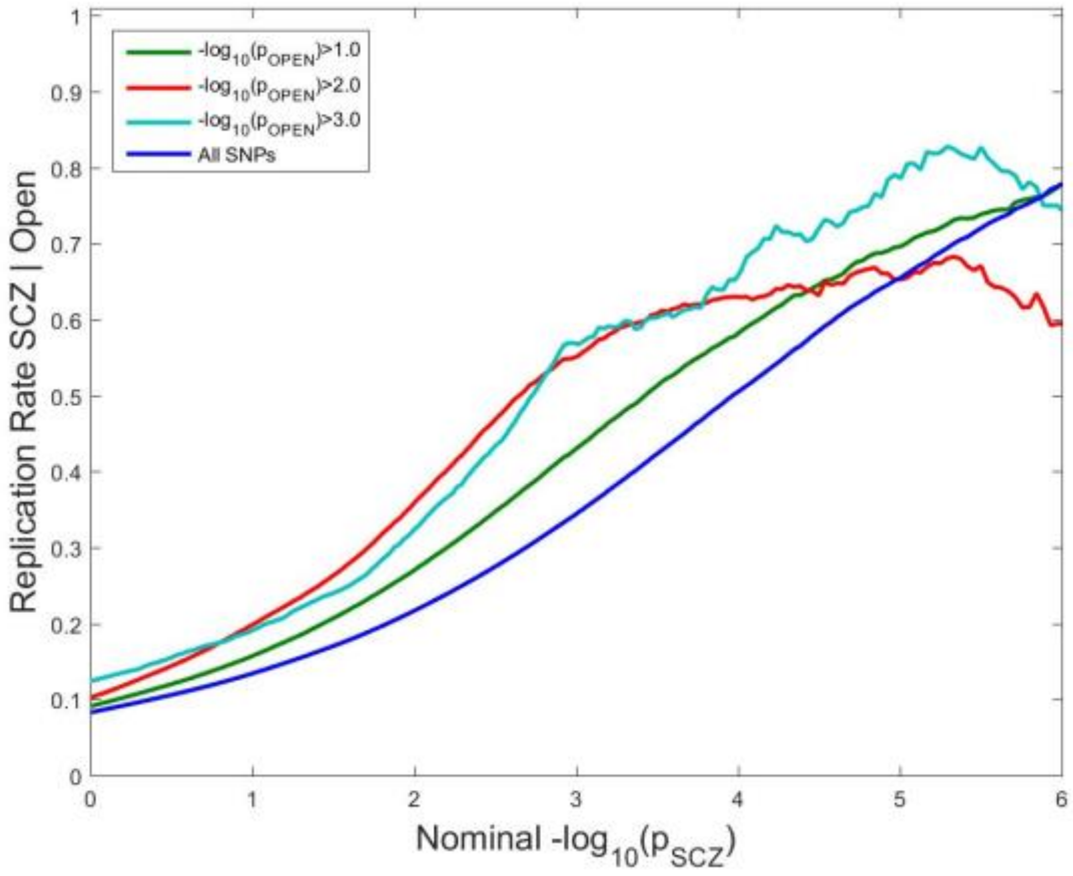

Supplementary figure 9. Cumulative replication plot showing the average rate of replication ($p < 0.05$) within schizophrenia (SCZ) substudies for a given p-value threshold demonstrates that pleiotropic enriched SNP categories replicate at a higher rate in independent SCZ samples, for SCZ conditioned on openness (OPEN). The vertical intercept is the overall replication rate per category. Analysis is based on split half method of the 52 PGC SCZ substudies.

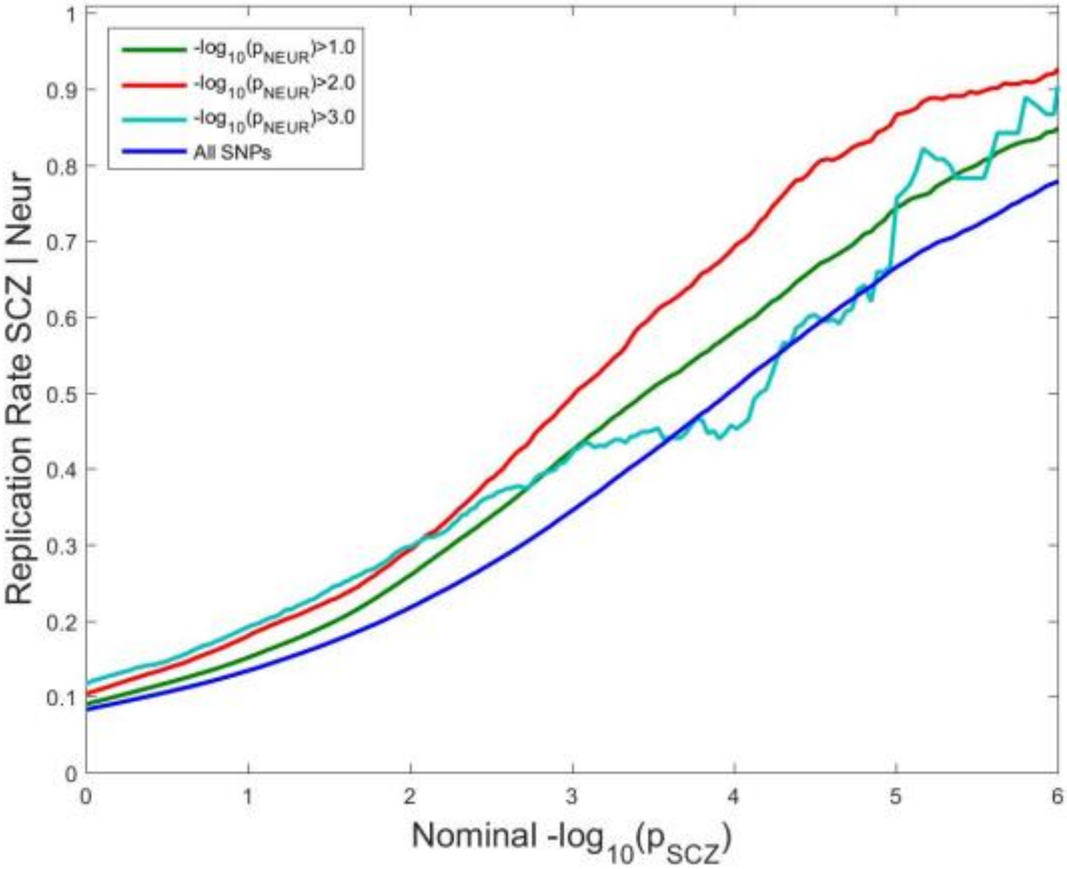

Supplementary figure 10. Cumulative replication plot showing the average rate of replication ($p < 0.05$) within schizophrenia (SCZ) substudies for a given p-value threshold demonstrates that pleiotropic enriched SNP categories replicate at a higher rate in independent SCZ samples, for SCZ conditioned on neuroticism (NEUR), although this is not evident for the category $-\log_{10}(p_{NEUR}) \geq 3$). The vertical intercept is the overall replication rate per category. Analysis is based on split half method of the 52 PGC SCZ substudies.

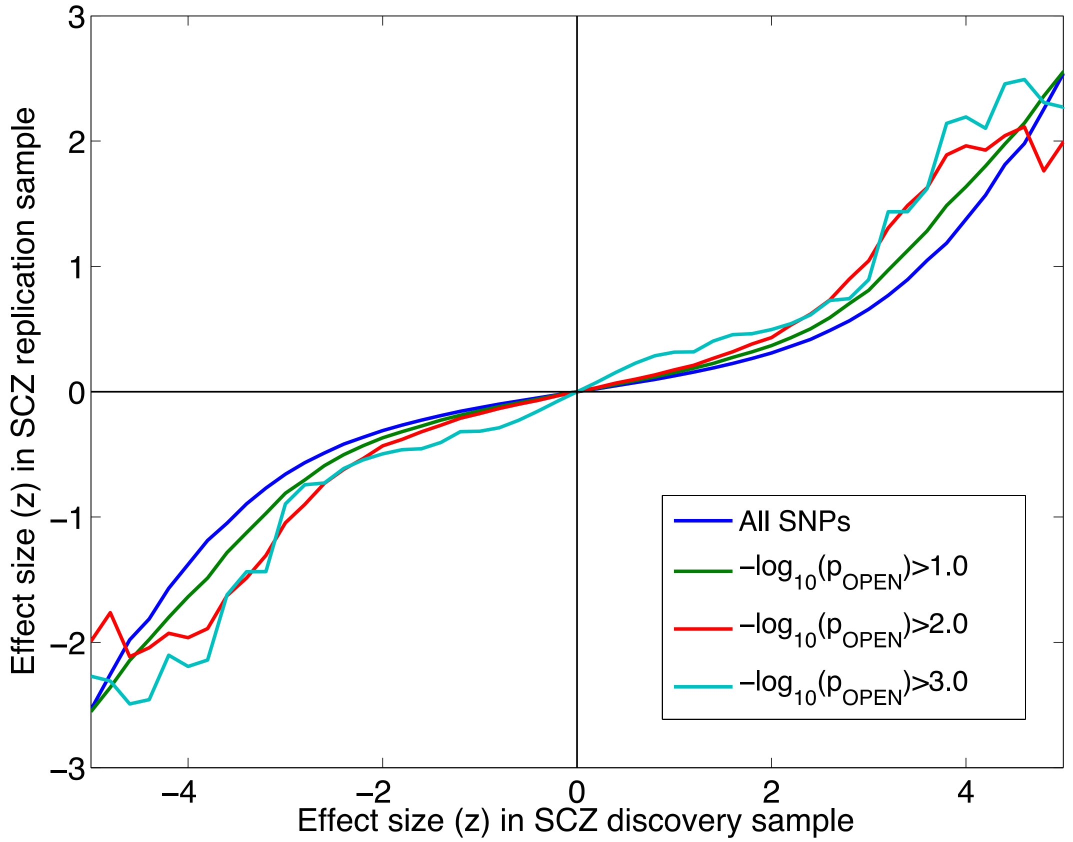

Supplementary Figure 11. Z-score-z-score plot demonstrates that the empirical replication z-scores closely match the expected a posteriori effect sizes of schizophrenia and depend upon genetic overlap with openness (OPEN). Analysis is based on split half method of the 52 PGC SCZ substudies.

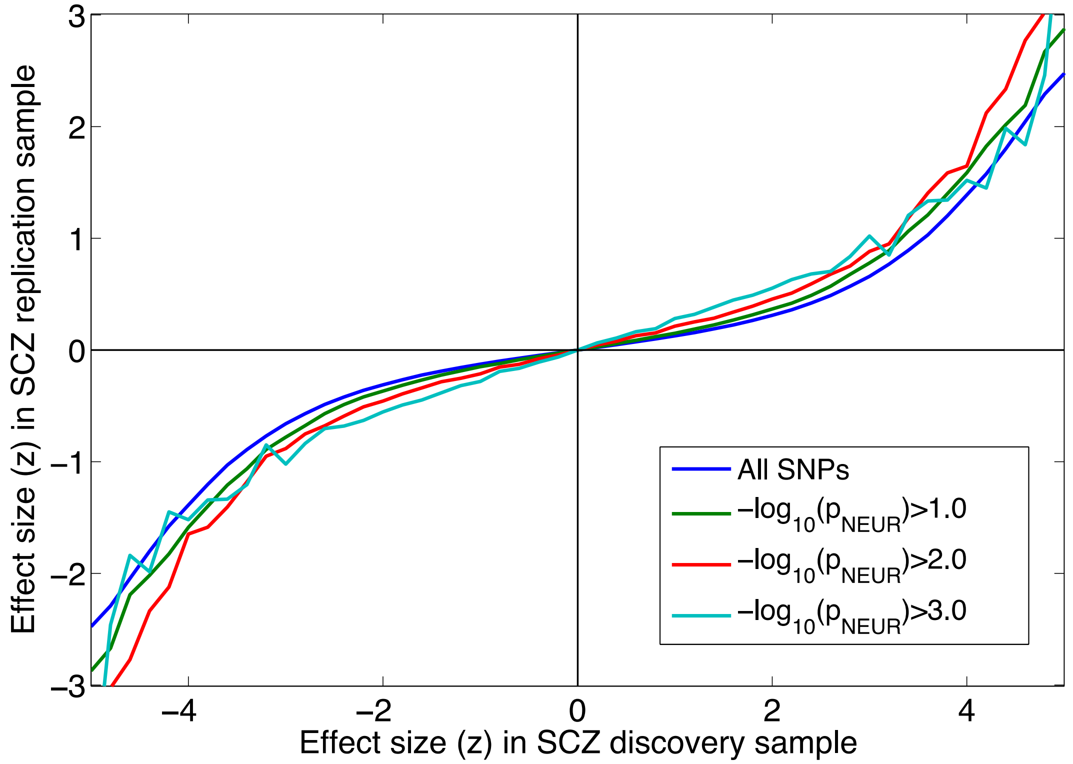

Supplementary Figure 12. Z-score-z-score plot demonstrates that the empirical replication z-scores closely match the expected a posteriori effect sizes of schizophrenia and correlate with genetic overlap with neuroticism (NEUR). Analysis is based on split half method of the 52 PGC SCZ substudies.
